# (3*S*,11*Z*)-14,16-Dihy­droxy-3-methyl-3,4,5,6,9,10-hexa­hydro-1*H*-2-benz­oxacyclo­tetra­decine-1,7(8*H*)-dione (*cis*-zearalenone): a redetermination

**DOI:** 10.1107/S1600536812002735

**Published:** 2012-02-24

**Authors:** Robert Köppen, Juliane Riedel, Franziska Emmerling, Matthias Koch

**Affiliations:** a BAM Federal Institute for Materials Research and Testing, Department of Analytical Chemistry, Reference Materials, Richard-Willstätter-Strasse 11, D-12489 Berlin-Adlershof, Germany

## Abstract

The title compound, also known as *cis*-zearalenone (*cis*-ZEN), C_18_H_22_O_5_, has already been reported elsewhere [Griffin *et al.* (1981 ▶). *ACA Ser.* **29**, 35], but no atomic coordinates are publicly available. The mol­ecule is of inter­est with respect to its toxicity. In the crystal, intra­molecular O—H⋯O hydrogen bonds stabilize the mol­ecular conformation, while inter­molecular O—H⋯O hydrogen bonds link the mol­ecules to form infinite chains along the [110] and [1-10] directions. The absolute configuration has been assigned by reference to an unchanging chiral centre in the synthetic procedure.

## Related literature

For the crystal structures of *trans*-zearalenone (*trans*-ZEN) and zearalenol, see: Gelo-Pujić *et al.* (1994 ▶) and Zhao *et al.* (2008 ▶). For more detailed information about *trans*-ZEN and its metabolites, see: Urry *et al.* (1966 ▶) and Zinedine *et al.* (2007 ▶). 
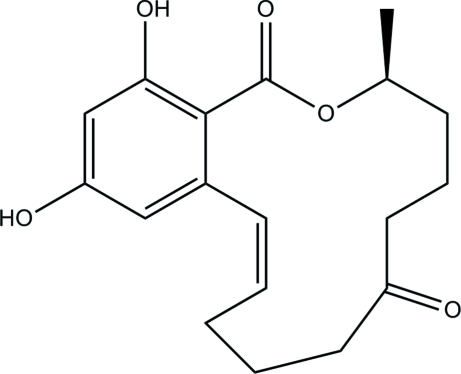


## Experimental

### 

#### Crystal data

C_18_H_22_O_5_
*M* *_r_* = 318.36
Monoclinic, 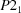

*a* = 5.677 (3) Å
*b* = 9.186 (4) Å
*c* = 16.531 (7) Å
β = 98.91 (3)°
*V* = 851.7 (7) Å^3^
*Z* = 2
Mo *K*α radiation
μ = 0.09 mm^−1^
*T* = 296 K
0.3 × 0.1 × 0.05 mm

#### Data collection

Bruker APEX CCD area-detector diffractometer
Absorption correction: ψ scan (*SHELXTL*; Sheldrick, 2008 ▶) *T*_min_ = 0.21, *T*_max_ = 0.28
20096 measured reflections
1976 independent reflections
1014 reflections with *I* > 2σ(*I*)
*R*_int_ = 0.101

#### Refinement

*R*[*F* ^2^ > 2σ(*F* ^2^)] = 0.038
*wR*(*F* ^2^) = 0.124
*S* = 0.87
1976 reflections
209 parameters
1 restraint
H-atom parameters constrained
Δρ_max_ = 0.14 e Å^−3^
Δρ_min_ = −0.11 e Å^−3^

### 

Data collection: *SMART* (Bruker, 2001 ▶); cell refinement: *SAINT* (Bruker, 2001 ▶); data reduction: *SAINT*; program(s) used to solve structure: *SHELXS97* (Sheldrick, 2008 ▶); program(s) used to refine structure: *SHELXL97* (Sheldrick, 2008 ▶); molecular graphics: *SHELXTL* (Bruker, 2001 ▶) and *ORTEPIII* (Burnett & Johnson, 1996 ▶); software used to prepare material for publication: *SHELXTL*.

## Supplementary Material

Crystal structure: contains datablock(s) I, global. DOI: 10.1107/S1600536812002735/bg2443sup1.cif

Structure factors: contains datablock(s) I. DOI: 10.1107/S1600536812002735/bg2443Isup2.hkl

Supplementary material file. DOI: 10.1107/S1600536812002735/bg2443Isup3.mol

Supplementary material file. DOI: 10.1107/S1600536812002735/bg2443Isup4.cml

Additional supplementary materials:  crystallographic information; 3D view; checkCIF report

## Figures and Tables

**Table 1:** Hydrogen-bond geometry (Å, °)

*D*—H⋯*A*	*D*—H	H⋯*A*	*D*⋯*A*	*D*—H⋯*A*
O5—H22⋯O2	0.82	1.84	2.569 (5)	148
O4—H20⋯O3^i^	0.82	2.01	2.824 (5)	169

Symmetry code: (i) 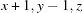.

**Fig. 1.**
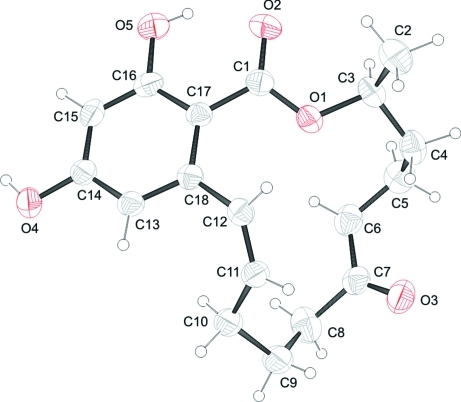
A molecular representation of the title compound with atomic labelling (30% probability displacement ellipsoids).

**Fig. 2.**
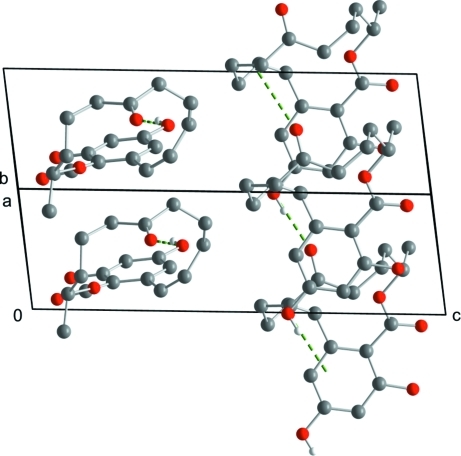
View of the unit cell of the title compound along [-110], showing the hydrogen-bonded chains along the [110] and [-110] directions. Hydrogen bonds are drawn as dashed green lines.

## supplementary crystallographic information

### Comment

Zearalenone (ZEN) is an estrogenic secondary fungal metabolite produced by some species from the genus *Fusarium*, such as *F. graminearum* (teleomorph *Gibberella zeae*) and *F. culmorum* on a variety of cereals. ZEN is one of the worldwide most common mycotoxins in cereal grains and animal feeds and, consequently, humans and animals are at risk of being exposed to ZEN by consuming contaminated food products and feeds.

In chemical terms, zearalenone belongs to the group of resorcyclic acid lactones. Due to the ethylenic double bond between C_11_ and C_12_ in the lactone ring ZEN can exist in two stereoisomeric forms: *cis* and *trans*. From mycelia of the fungus *F. graminearum* only *trans*-ZEN could be isolated and its structure was elucidated using classical chemical, NMR and mass spectrometric analysis (Urry *et al.* 1966). This finding, which was confirmed also by other studies, led to the assumption that in the ZEN production by the fungi an isomer specific biosynthetic pathway is involved. According to IUPAC the name zearalenone is a synonym only for the pure (3*S*,11*E*)-14,16-dihydroxy-3-methyl-3,4,5,6,9,10-hexahydro-1*H*-2-benzoxacyclotetradecine-1,7(8*H*)-dione (= *trans*-ZEN, CAS: 17924–92–4) and describes not the isomeric mixture of *cis*- and *trans*-ZEN. Therefore, worldwide all established maximum levels for ZEN in food and feed apply only to *trans*-ZEN. However, the absorption of (ultraviolet) light induces isomerization from *trans*- to the more stable *cis*-ZEN, so that at presence of any ZEN contamination both isomers can occur. Only very little is known about the occurrence, fate and risks associated with *cis*-ZEN entering the food chain. This causes a major problem for the official control of foodstuffs and consumer protection. Most of the various analytical methods for the determination of ZEN in food and feed, including the official methods (*e.g.*, ASU (german: "Amtliche Sammlung von Untersuchungsverfahren") according to paragraph 64 of the LFGB (german: "Lebensmittel-, Bedarfsgegenstände- und Futtermittelgesetzbuch")) are not able to distinguish between the two ZEN isomers. Hence, depending on the chromatographic separation this could potentially lead to "false positive" or "false negative" results and therefore to enormous public health or economic consequences. The compound crystallizes in the monoclinic space group *P*2_1_.

The molecular structure of the compound had already been reported elsewhere (Griffin *et al.*, 1981; CCDC code: ZEARLN) but no atomic coordinates were made publicly available at the time, for what the present redetermination was attempted.

The atom-labeling scheme is shown in Fig. 1. The absolute configuration could not be defined confidently based on the single-crystal diffraction data. It should be noted that a light induced *cis*-/*trans*- isomerization of pure (stereochemical defined) *trans*-ZEN proceeds under retention of the stereochemical sense at C_3_. The isomeric purity of the title compound was confirmed by ^1^H-NMR, HPLC-DAD and –MS/MS data. Besides the intramolecular hydrogen bonds between O5—H22 and O2 (not shown in Fig. 2), each molecule is connected to two adjacent molecules *via* intermolecular hydrogen bonds (see dashed green bonds in Fig. 2). As a result infinite chains are formed along [110] and [-110] direction (see Fig. 2).

### Experimental

25 mg (78.5 µmol) of pure *trans*-ZEN (purity 99.8%), obtained from AppliChem GmbH (Darmstadt, Germany), were dissolved in Acetonitrile (18 ml) and irradiated at 23 °C for 8 h with ultraviolet light (λ=350 nm, Universal UV-Lampe, Typ TL-900; CAMAG (Muttenz, Switzerland)). Separation of *cis*-ZEN from the reaction mixture was carried out by semi-preparative HPLC (Phenomenex Gemini-NX C_18_ column; 150x2 mm, 3 µm) with ACN:H_2_O (38:62, v:v) as eluent. The purity of the isolated white powder (yield: 16 mg (64%)) was determined to be ≥95% by analytical HPLC-FLD. In addition, ^1^H-NMR and HPLC-MS/MS have also been used to identify *cis*-ZEN and to evaluate its purity. For structural identification colorless crystals were grown by slow solvent evaporation in absence of light at ambient temperature as detailed below. In a 1.5 ml HPLC glass vial 5.0 mg (18.5 µmol) of purified *cis*-ZEN were weighed in and dissolved in 0.5 ml dichloromethane. Afterwards, n-hexane (1.0 ml) was added to the incipient precipitation point and then the solution was set aside at room temperature for 72 h in the dark to evaporate slowly. The title compound crystallized as colorless plates.

### Refinement

All the H-Atoms were found in a Difference Map but positioned geometrically and refined using a riding model with d(C—H) = 0.93 Å, *U*_iso_=1.2*U*_eq_ (C) for aromatic 0.98 Å, *U*_iso_ = 1.2*U*_eq_ (C) for CH, 0.97 Å, *U*_iso_ = 1.2*U*_eq_ (C) for CH_2_, 0.96 Å, *U*_iso_ = 1.5*U*_eq_ (C) for CH_3_ atoms, and 0.82 Å, *U*_iso_ = 1.5*U*_eq_ (C) for hydroxyl groups. In the absence of significant anomalous dispersion effects, Friedel pairs were merged.

### Crystal data

**Table:**

C_18_H_22_O_5_	*F*(000) = 340
*M**_r_* = 318.36	*D*_x_ = 1.241 Mg m^−^^3^
Monoclinic, *P*2_1_	Mo *K*α radiation, λ = 0.71073 Å
Hall symbol: P 2yb	Cell parameters from 56 reflections
*a* = 5.677 (3) Å	θ = 4–25°
*b* = 9.186 (4) Å	µ = 0.09 mm^−^^1^
*c* = 16.531 (7) Å	*T* = 296 K
β = 98.91 (3)°	Plate, colourless
*V* = 851.7 (7) Å^3^	0.3 × 0.1 × 0.05 mm
*Z* = 2	

### Data collection

**Table:**

Bruker APEX CCD area-detector diffractometer	1976 independent reflections
Radiation source: fine-focus sealed tube	1014 reflections with *I* > 2σ(*I*)
Graphite monochromator	*R*_int_ = 0.101
ω/2θ scans	θ_max_ = 27.0°, θ_min_ = 1.3°
Absorption correction: ψ scan (*SHELXTL*; Sheldrick, 2008)	*h* = −7→7
*T*_min_ = 0.21, *T*_max_ = 0.28	*k* = −11→11
20096 measured reflections	*l* = −21→21

### Refinement

**Table:**

Refinement on *F*^2^	Primary atom site location: structure-invariant direct methods
Least-squares matrix: full	Secondary atom site location: difference Fourier map
*R*[*F*^2^ > 2σ(*F*^2^)] = 0.038	Hydrogen site location: inferred from neighbouring sites
*wR*(*F*^2^) = 0.124	H-atom parameters constrained
*S* = 0.87	*w* = 1/[σ^2^(*F*_o_^2^) + (0.0678*P*)^2^] where *P* = (*F*_o_^2^ + 2*F*_c_^2^)/3
1976 reflections	(Δ/σ)_max_ < 0.001
209 parameters	Δρ_max_ = 0.14 e Å^−^^3^
1 restraint	Δρ_min_ = −0.11 e Å^−^^3^

### Special details

**Table:**

Geometry. All e.s.d.'s (except the e.s.d. in the dihedral angle between two l.s. planes) are estimated using the full covariance matrix. The cell e.s.d.'s are taken into account individually in the estimation of e.s.d.'s in distances, angles and torsion angles; correlations between e.s.d.'s in cell parameters are only used when they are defined by crystal symmetry. An approximate (isotropic) treatment of cell e.s.d.'s is used for estimating e.s.d.'s involving l.s. planes.
Refinement. Refinement of *F*^2^ against ALL reflections. The weighted *R*-factor *wR* and goodness of fit *S* are based on *F*^2^, conventional *R*-factors *R* are based on *F*, with *F* set to zero for negative *F*^2^. The threshold expression of *F*^2^ > σ(*F*^2^) is used only for calculating *R*-factors(gt) *etc*. and is not relevant to the choice of reflections for refinement. *R*-factors based on *F*^2^ are statistically about twice as large as those based on *F*, and *R*- factors based on ALL data will be even larger.

### Fractional atomic coordinates and isotropic or equivalent isotropic displacement parameters (Å^2^)

**Table:**

	*x*	*y*	*z*	*U*_iso_*/*U*_eq_	
O1	0.7716 (4)	0.4105 (3)	0.15608 (15)	0.0636 (7)	
O2	0.8395 (5)	0.2441 (3)	0.06309 (17)	0.0829 (9)	
O3	0.7827 (6)	0.8046 (4)	0.30820 (19)	0.0888 (10)	
O4	1.5275 (5)	0.0183 (3)	0.38175 (16)	0.0758 (9)	
H20	1.6166	−0.0360	0.3613	0.114*	
O5	1.1936 (6)	0.0703 (4)	0.10159 (17)	0.0871 (9)	
H22	1.0861	0.1128	0.0719	0.131*	
C1	0.8686 (7)	0.2863 (5)	0.1351 (3)	0.0606 (10)	
C2	0.3806 (8)	0.4346 (7)	0.0708 (3)	0.0937 (15)	
H4	0.3069	0.4314	0.1192	0.141*	
H2	0.3917	0.3377	0.0498	0.141*	
H3	0.2863	0.4938	0.0301	0.141*	
C3	0.6293 (7)	0.4994 (5)	0.0915 (2)	0.0691 (12)	
H1	0.7070	0.5021	0.0426	0.083*	
C4	0.6229 (8)	0.6532 (5)	0.1284 (3)	0.0783 (13)	
H5	0.5046	0.7097	0.0930	0.094*	
H6	0.5681	0.6445	0.1809	0.094*	
C5	0.8534 (9)	0.7391 (6)	0.1412 (3)	0.0888 (14)	
H8	0.8148	0.8417	0.1434	0.107*	
H7	0.9330	0.7245	0.0939	0.107*	
C6	1.0269 (8)	0.7005 (6)	0.2177 (3)	0.0814 (13)	
H9	1.0458	0.5956	0.2189	0.098*	
H10	1.1806	0.7419	0.2120	0.098*	
C7	0.9691 (9)	0.7467 (4)	0.2989 (3)	0.0684 (11)	
C8	1.1633 (9)	0.7214 (6)	0.3731 (3)	0.0950 (15)	
H12	1.2920	0.6674	0.3547	0.114*	
H11	1.2269	0.8154	0.3922	0.114*	
C9	1.0909 (10)	0.6422 (5)	0.4445 (3)	0.0876 (14)	
H13	0.9359	0.6776	0.4533	0.105*	
H14	1.2040	0.6649	0.4931	0.105*	
C10	1.0780 (9)	0.4767 (4)	0.4340 (3)	0.0770 (13)	
H16	1.2174	0.4438	0.4119	0.092*	
H15	1.0820	0.4318	0.4873	0.092*	
C11	0.8601 (7)	0.4273 (4)	0.3791 (2)	0.0613 (10)	
H17	0.7185	0.4688	0.3897	0.074*	
C12	0.8360 (7)	0.3329 (4)	0.3171 (2)	0.0549 (10)	
H18	0.6828	0.3230	0.2881	0.066*	
C13	1.1930 (7)	0.1716 (4)	0.3452 (2)	0.0578 (10)	
H19	1.1928	0.1882	0.4007	0.069*	
C14	1.3643 (7)	0.0777 (4)	0.3214 (2)	0.0590 (10)	
C15	1.3633 (7)	0.0455 (4)	0.2402 (3)	0.0643 (11)	
H21	1.4758	−0.0179	0.2246	0.077*	
C16	1.1909 (8)	0.1094 (4)	0.1815 (2)	0.0648 (11)	
C17	1.0217 (7)	0.2105 (4)	0.2032 (2)	0.0535 (9)	
C18	1.0224 (6)	0.2411 (4)	0.2881 (2)	0.0516 (9)	

### Atomic displacement parameters (Å^2^)

**Table:**

	*U*^11^	*U*^22^	*U*^33^	*U*^12^	*U*^13^	*U*^23^
O1	0.0711 (17)	0.0634 (17)	0.0536 (15)	0.0035 (16)	0.0008 (12)	0.0067 (14)
O2	0.097 (2)	0.097 (2)	0.0508 (17)	0.0073 (19)	−0.0019 (15)	−0.0117 (16)
O3	0.093 (2)	0.078 (2)	0.097 (2)	0.024 (2)	0.0210 (19)	0.0020 (18)
O4	0.0816 (19)	0.0612 (18)	0.0781 (19)	0.0113 (15)	−0.0079 (15)	0.0037 (15)
O5	0.110 (2)	0.088 (2)	0.0641 (18)	0.018 (2)	0.0173 (16)	−0.0149 (16)
C1	0.059 (2)	0.064 (3)	0.059 (3)	−0.004 (2)	0.009 (2)	−0.002 (2)
C2	0.072 (3)	0.110 (4)	0.093 (3)	−0.001 (3)	−0.007 (2)	0.004 (3)
C3	0.063 (3)	0.082 (3)	0.059 (2)	0.002 (2)	0.0022 (19)	0.015 (2)
C4	0.077 (3)	0.081 (3)	0.076 (3)	0.012 (3)	0.012 (2)	0.025 (3)
C5	0.101 (4)	0.080 (3)	0.089 (3)	−0.001 (3)	0.028 (3)	0.014 (3)
C6	0.075 (3)	0.065 (3)	0.103 (4)	−0.003 (2)	0.009 (3)	−0.004 (3)
C7	0.085 (3)	0.045 (2)	0.076 (3)	−0.005 (2)	0.015 (2)	0.002 (2)
C8	0.084 (3)	0.084 (3)	0.109 (4)	−0.013 (3)	−0.009 (3)	0.014 (3)
C9	0.130 (4)	0.052 (2)	0.074 (3)	−0.001 (3)	−0.005 (3)	−0.006 (2)
C10	0.112 (4)	0.049 (2)	0.065 (3)	−0.001 (2)	−0.001 (2)	−0.001 (2)
C11	0.079 (3)	0.053 (2)	0.053 (2)	0.002 (2)	0.0143 (19)	0.002 (2)
C12	0.058 (2)	0.054 (2)	0.053 (2)	−0.0043 (19)	0.0102 (18)	0.0073 (19)
C13	0.075 (3)	0.047 (2)	0.051 (2)	−0.007 (2)	0.009 (2)	−0.0010 (18)
C14	0.072 (3)	0.043 (2)	0.061 (3)	−0.009 (2)	0.005 (2)	−0.001 (2)
C15	0.069 (3)	0.047 (2)	0.077 (3)	0.006 (2)	0.011 (2)	−0.003 (2)
C16	0.084 (3)	0.055 (3)	0.059 (3)	−0.007 (2)	0.021 (2)	−0.011 (2)
C17	0.060 (2)	0.049 (2)	0.051 (2)	−0.0067 (19)	0.0063 (18)	−0.0027 (17)
C18	0.060 (2)	0.042 (2)	0.054 (2)	−0.0102 (19)	0.0100 (18)	0.0021 (18)

### Geometric parameters (Å, º)

**Table:**

O1—C1	1.336 (5)	C6—H10	0.9700
O1—C3	1.480 (4)	C7—C8	1.535 (6)
O2—C1	1.238 (4)	C8—C9	1.497 (7)
O3—C7	1.215 (5)	C8—H12	0.9700
O4—C14	1.366 (4)	C8—H11	0.9700
O4—H20	0.8200	C9—C10	1.531 (6)
O5—C16	1.371 (4)	C9—H13	0.9700
O5—H22	0.8200	C9—H14	0.9700
C1—C17	1.485 (5)	C10—C11	1.487 (6)
C2—C3	1.522 (6)	C10—H16	0.9700
C2—H4	0.9600	C10—H15	0.9700
C2—H2	0.9600	C11—C12	1.334 (5)
C2—H3	0.9600	C11—H17	0.9300
C3—C4	1.542 (6)	C12—C18	1.488 (5)
C3—H1	0.9800	C12—H18	0.9300
C4—C5	1.514 (6)	C13—C18	1.398 (5)
C4—H5	0.9700	C13—C14	1.401 (5)
C4—H6	0.9700	C13—H19	0.9300
C5—C6	1.521 (6)	C14—C15	1.373 (5)
C5—H8	0.9700	C15—C16	1.397 (5)
C5—H7	0.9700	C15—H21	0.9300
C6—C7	1.491 (6)	C16—C17	1.422 (5)
C6—H9	0.9700	C17—C18	1.432 (5)
			
C1—O1—C3	119.0 (3)	C7—C8—H12	108.1
C14—O4—H20	109.5	C9—C8—H11	108.1
C16—O5—H22	109.5	C7—C8—H11	108.1
O2—C1—O1	121.2 (4)	H12—C8—H11	107.3
O2—C1—C17	123.9 (4)	C8—C9—C10	114.0 (4)
O1—C1—C17	114.9 (3)	C8—C9—H13	108.7
C3—C2—H4	109.5	C10—C9—H13	108.7
C3—C2—H2	109.5	C8—C9—H14	108.7
H4—C2—H2	109.5	C10—C9—H14	108.7
C3—C2—H3	109.5	H13—C9—H14	107.6
H4—C2—H3	109.5	C11—C10—C9	113.1 (4)
H2—C2—H3	109.5	C11—C10—H16	109.0
O1—C3—C2	109.3 (3)	C9—C10—H16	109.0
O1—C3—C4	105.3 (3)	C11—C10—H15	109.0
C2—C3—C4	111.7 (4)	C9—C10—H15	109.0
O1—C3—H1	110.1	H16—C10—H15	107.8
C2—C3—H1	110.1	C12—C11—C10	130.1 (4)
C4—C3—H1	110.1	C12—C11—H17	115.0
C5—C4—C3	117.4 (4)	C10—C11—H17	115.0
C5—C4—H5	108.0	C11—C12—C18	128.5 (4)
C3—C4—H5	108.0	C11—C12—H18	115.8
C5—C4—H6	108.0	C18—C12—H18	115.8
C3—C4—H6	108.0	C18—C13—C14	122.0 (3)
H5—C4—H6	107.2	C18—C13—H19	119.0
C4—C5—C6	115.4 (4)	C14—C13—H19	119.0
C4—C5—H8	108.4	O4—C14—C15	121.8 (4)
C6—C5—H8	108.4	O4—C14—C13	117.5 (3)
C4—C5—H7	108.4	C15—C14—C13	120.6 (4)
C6—C5—H7	108.4	C14—C15—C16	119.0 (4)
H8—C5—H7	107.5	C14—C15—H21	120.5
C7—C6—C5	118.6 (4)	C16—C15—H21	120.5
C7—C6—H9	107.7	O5—C16—C15	116.7 (4)
C5—C6—H9	107.7	O5—C16—C17	121.5 (4)
C7—C6—H10	107.7	C15—C16—C17	121.8 (4)
C5—C6—H10	107.7	C16—C17—C18	118.5 (3)
H9—C6—H10	107.1	C16—C17—C1	117.0 (3)
O3—C7—C6	123.8 (4)	C18—C17—C1	124.3 (3)
O3—C7—C8	119.8 (4)	C13—C18—C17	118.0 (3)
C6—C7—C8	116.4 (4)	C13—C18—C12	119.6 (3)
C9—C8—C7	116.9 (4)	C17—C18—C12	122.2 (3)
C9—C8—H12	108.1		
			
C3—O1—C1—O2	−0.7 (5)	C13—C14—C15—C16	0.9 (6)
C3—O1—C1—C17	175.9 (3)	C14—C15—C16—O5	−178.2 (3)
C1—O1—C3—C2	79.8 (4)	C14—C15—C16—C17	2.2 (6)
C1—O1—C3—C4	−160.1 (3)	O5—C16—C17—C18	177.2 (3)
O1—C3—C4—C5	69.7 (4)	C15—C16—C17—C18	−3.2 (6)
C2—C3—C4—C5	−171.8 (4)	O5—C16—C17—C1	−7.7 (5)
C3—C4—C5—C6	−80.4 (5)	C15—C16—C17—C1	171.9 (4)
C4—C5—C6—C7	−72.6 (6)	O2—C1—C17—C16	17.5 (6)
C5—C6—C7—O3	5.5 (6)	O1—C1—C17—C16	−159.0 (3)
C5—C6—C7—C8	−172.9 (4)	O2—C1—C17—C18	−167.7 (4)
O3—C7—C8—C9	53.2 (6)	O1—C1—C17—C18	15.8 (5)
C6—C7—C8—C9	−128.3 (5)	C14—C13—C18—C17	1.9 (5)
C7—C8—C9—C10	80.1 (6)	C14—C13—C18—C12	176.4 (3)
C8—C9—C10—C11	−77.0 (6)	C16—C17—C18—C13	1.1 (5)
C9—C10—C11—C12	132.8 (5)	C1—C17—C18—C13	−173.6 (3)
C10—C11—C12—C18	4.5 (7)	C16—C17—C18—C12	−173.3 (3)
C18—C13—C14—O4	178.2 (3)	C1—C17—C18—C12	12.0 (5)
C18—C13—C14—C15	−3.0 (6)	C11—C12—C18—C13	41.9 (6)
O4—C14—C15—C16	179.7 (3)	C11—C12—C18—C17	−143.8 (4)

### Hydrogen-bond geometry (Å, º)

**Table:**

*D*—H···*A*	*D*—H	H···*A*	*D*···*A*	*D*—H···*A*
O5—H22···O2	0.82	1.84	2.569 (5)	148
O4—H20···O3^i^	0.82	2.01	2.824 (5)	169

Symmetry code: (i) *x*+1, *y*−1, *z*.
